# Editorial to “Revitalizing brain perfusion: Unveiling advancements through rhythm control strategies in atrial fibrillation—A systematic review”

**DOI:** 10.1002/joa3.13085

**Published:** 2024-05-28

**Authors:** Yoshimori An

**Affiliations:** ^1^ Department of Cardiology Osaka Saiseikai Noe Hospital Osaka Japan

**Keywords:** atrial fibrillation, brain perfusion, cognitive impairment, dementia, rhythm control

This is an editorial comment on the article presented by Rasti et al.^1^ as a systematic review focusing on published data regarding the impact of rhythm control therapy for atrial fibrillation (AF) on brain perfusion.

AF is one of the most common cardiac arrhythmias that increases the risk of stroke and death. As the prevalence of AF increases with age, it has been exhibiting an upward trend globally, with a worldwide prevalence ranging between 3 and 6 million individuals—which is projected to reach 6–16 million by 2050.^2^ Dementia is a condition that leads to a loss of cognitive function, affecting daily life activities. Alzheimer's disease and vascular dementia represent the two main subvarieties of dementia. Studies have shown that AF is independently associated with cognitive decline. The proposed pathophysiological mechanisms include silent ischemic or hemorrhagic cerebral microinfarction, or impaired cerebral blood flow. Recently, it has been shown that AF is associated with a risk of dementia, independent of clinical stroke.^3^ Brain perfusion may be reduced in patients with AF, which may contribute to cognitive impairment. Theoretically, rhythm control therapy including catheter ablation (CA) may positively affect brain perfusion in patients with AF, because the restoration of the sinus rhythm may increase cardiac output. However, the evidence supporting this notion is not yet well established, and research on this topic is ongoing.

In a recent issue of *Journal of Arrhythmia*, Rasti et al. presented a systematic review on the topic that included articles from Scopus, PubMed, Cochrane Reviews, ProQuest, and EBSCOhost databases, searched from their respective inceptions until April 30, 2023.^1^ A total of 10 studies (436 patients with AF) that met their criteria were reviewed. They found that restoring the sinus rhythm enhanced brain perfusion in 8 of the 10 studies. The authors therefore concluded that successful control of the AF rhythm enhances brain perfusion and mitigates cognitive decline. They reviewed published data on the subject and provided valuable information for physicians. Their conclusions are promising and helpful but must be interpreted cautiously. A wide variety of methods exist for both achieving rhythm control and measuring the outcomes. One of the studies reviewed used a pharmacological approach (amiodarone), six used electrical cardioversion, and three used CA (pulmonary vein isolation by radiofrequency ablation or cryoballoon and atrioventricular node ablation after pacemaker implantation). The maintenance rate of sinus rhythm differs depending on the method used to control it, thus exerting a range of effects on brain perfusion outcomes. Regarding the outcome measures, the methods used to evaluate brain perfusion can be categorized as direct and indirect. In seven of the 10 studies included in the review article, cerebral blood flow (CBF) was measured using a direct method, which was quantified as the volume of blood passing through brain tissue per unit of time (typically expressed as mL/100 g of brain tissue per minute). The other three studies used indirect methods including cerebral tissue oxygen saturation (SctO_2_; two studies) and tissue hemoglobin index (THI; one study). Differences in outcome measures should be recognized when interpreting the results of each study included in this review. Study heterogeneity likely contributed significantly to differences in the results.

Cognitive function was also evaluated before and after rhythm control for AF in 4 of the 10 studies investigated in their review article. Two of these reported a positive effect of rhythm control on cognitive function, whereas the other two claimed a negative effect. The methods used in the two studies with positive results were pulmonary vein isolation by radiofrequency ablation or cryoballoon and atrioventricular node ablation following pacemaker implantation. The other two studies with negative results used electrical cardioversion. It should be recognized that the methods and timings used to evaluate cognitive function differed among these four studies as well. Regarding the effect of CA on cognitive function, the influence of periprocedural cerebral emboli and anesthesia during the ablation procedure should be considered. The results of cognitive function tests vary depending on when the patient is evaluated following CA. Jin et al. demonstrated that Montreal Cognitive Assessment scores improved 12 months after CA.^4^ Meanwhile, subclinical cerebral emboli in the subacute phase, such as within 3 months after CA, may worsen cognitive impairment. Moreover, the effect of rhythm control on dementia has recently been reported to differ depending on the disease subtype. A recent meta‐analysis demonstrated that those who received CA had a lower risk of Alzheimer's disease (hazard ratio, 0.78 [95% confidential interval: 0.66–0.92]; *p* < .001) compared with the non‐CA group.^5^ However, there was no statistically significant difference in the risk of vascular dementia between the patient groups. This may be because the protective effect of sinus rhythm maintenance on vascular dementia is counteracted by potential blood clot formation and undetected brain damage after the procedure.

Considering the lack of established data on the subject, further studies are warranted to clarify the true impact of rhythm control, including CA versus medical therapy, on cognitive function and the incidence of dementia. To mitigate the risk of selection bias, randomized trials are required to compare the effects of AF treatment strategies on cognition. The DIAL‐F case–control study (cognitive impairment in atrial fibrillation; unique identifier: NCT01816308) is currently ongoing and aims to compare the incidence of cognitive impairment between two groups of patients with AF (those undergoing catheter ablation for AF vs. those receiving antiarrhythmic drugs). Ongoing studies, including this trial, are expected to further clarify the link between AF and dementia. This will hopefully lead to treatments that slow the progression of dementia. The review article written by Rasti et al.^1^ helps to summarize what is known about this important topic and the issues involved.

## CONFLICT OF INTEREST STATEMENT

Authors declare no conflict of interests for this article.
